# Repeated talaporfin sodium photodynamic therapy for esophageal cancer: safety and efficacy

**DOI:** 10.1007/s10388-021-00853-x

**Published:** 2021-06-09

**Authors:** Masashi Tamaoki, Akira Yokoyama, Takahiro Horimatsu, Kenshiro Hirohashi, Yusuke Amanuma, Hirokazu Higuchi, Yosuke Mitani, Masahiro Yoshioka, Shinya Ohashi, Manabu Muto

**Affiliations:** 1 Department of Therapeutic Oncology, Graduate School of Medicine, Kyoto University, 54 Kawahara-cho, Shogoin, Sakyo-ku, Kyoto, 606-8507 Japan; 2 Department of Clinical Trial Promotion, Chiba Cancer Center, Chiba, Japan; 3 Department of Medical Supply, Kyoto University Hospital, Kyoto, Japan; 4 Preemptive Medicine and Lifestyle Disease Research Center, Kyoto University Hospital, Kyoto, Japan

**Keywords:** Esophageal cancer, Salvage treatment, Photodynamic therapy

## Abstract

**Background:**

Talaporfin sodium photodynamic therapy (tPDT) is an effective salvage treatment for local failure after chemoradiotherapy for esophageal cancer. Repeated tPDT could also be indicated for local recurrence or residue after the first salvage tPDT. However, the safety and efficacy of repeated tPDT have not been elucidated.

**Methods:**

We reviewed 52 patients with esophageal cancer who were treated with the first tPDT at Kyoto University Hospital between October 2015 and April 2020.

**Results:**

Among 52 patients, repeated tPDT after the first tPDT was indicated for 13 patients (25%), of which six had residual tumor, four had local recurrence after complete response (CR) after the first tPDT at the primary site, and six had metachronous lesion. The total session of repeated tPDT was 25; 16 were for primary sites and nine were for metachronous sites. Among them, six patients (46.2%) achieved local (L)-CR and nine lesions (56.3%) achieved lesion L-CR. By session, 10 sessions (40%) achieved L-CR. There were no severe adverse events except for one patient; this patient showed grade 3 esophageal stenosis and perforation after the third tPDT on the same lesion that was previously treated with porfimer sodium photodynamic therapy four times.

**Conclusion:**

Repeated tPDT could be an effective and safe treatment for local failure even after salvage tPDT for esophageal cancer.

**Supplementary Information:**

The online version contains supplementary material available at 10.1007/s10388-021-00853-x.

## Introduction

Esophageal cancer is the sixth most common cause of cancer-related deaths worldwide [1]. Chemoradiotherapy (CRT) is a curative option for locally advanced esophageal cancers [2]. However, local failure is observed in 30–40% of patients treated with CRT [3, 4]. Therefore, salvage treatment for local failure is important for improving prognosis. Salvage surgery and endoscopic resection (ER) have been indicated for local failure after CRT. Salvage surgery is a highly curative treatment, but carries the risk of severe complications and treatment-related deaths [5–7]. ER is limited to superficial lesions [8–10]. Moreover, second-line chemotherapy with taxane or immune checkpoint inhibitors is the standard of care for recurrence after CRT, but the complete response (CR) rate is never high [11–13].

We previously reported that talaporfin sodium photodynamic therapy (tPDT) is an effective and safe treatment option for local failure after CRT [14]. The local CR (L-CR) rate of tPDT is 69–88.5% [14, 15]. No severe adverse events were observed. Salvage tPDT has a strong benefit for organ preservation and patients’ quality of life. However, the treatment strategy for local residue, local recurrence, and metachronous cancer after the first tPDT has not been evaluated. Local failure after the first tPDT could be practically treated by salvage surgery, chemotherapy, PDT, and ER. In patients that are not indicated for salvage surgery due to unfavorable systemic conditions, or not indicated for ER due to a deep invasion of the lesion, repeated tPDT is considered to be an option for local failure after the first tPDT. However, the efficacy and safety of repeated PDT remain unknown.

This study aimed to investigate the safety and efficacy of repeated tPDT as a salvage treatment for esophageal cancer.

## Patients and methods

### Patients

We retrospectively reviewed 52 patients with esophageal cancer who received the first tPDT at Kyoto University Hospital between October 2015 and April 2020.

The indications for repeated tPDT were as follows: (i) lesions limited to clinical T1 and T2; (ii) absence of any lymph node or distant metastasis; (iii) no indications for ER; and (iv) no indications for salvage surgery, or the refusal of patients to undergo salvage surgery.

This study was approved by the Kyoto University Hospital ethics committee (R0874), and we reviewed data recorded in electronic medical records.

### Staging

The clinical stage was determined according to the TNM classification of the International Union Against Cancer, 8th edition. The depth of the lesion was evaluated using endoscopic ultrasonography (EUS). When EUS was difficult to perform, we evaluated the depth of the lesion using conventional white-light images.

### Treatment

Talaporfin sodium (40 mg/m^2^) was administered intravenously. The lesion was irradiated with a diode laser at 664 nm wavelength 4 h after talaporfin sodium administration. The diode laser was delivered via a frontal light distributor through an endoscope. The fluence of the diode laser was set to 100 J/cm^2^, and the fluence rate was 150 mW/cm^2^. Multiple treatment fields were overlapped to cover the lesion according to its size. Endoscopic observation was performed on the following day. If a residual tumor was found, additional diode laser irradiation was performed. After the administration of talaporfin sodium, the patients stayed in a room maintained at less than 500 lx and avoided direct sun exposure for 2–4 weeks.

### Evaluation of efficacy and prognosis

The irradiated site was observed via endoscopic examination every 2–3 weeks after repeated tPDT until the disappearance of tPDT-induced ulcer. Local efficacy was classified via endoscopic evaluation as local CR (L-CR) and non local CR (non L-CR) at each evaluation. The criteria for L-CR were as follows: (1) disappearance of tPDT-induced ulcer and scar formation was confirmed; (2) no residual tumor was observed; (3) disappearance of cancer cells was assessed histologically as much as possible. If histological findings could not be confirmed for some reasons, we evaluated L-CR using endoscopic finding. When cancer was histologically detected after L-CR, it was defined as local recurrence and indicated salvage treatment.

L-CR rate was calculated as the percentage of patients whose all lesions treated by repeated tPDT achieved L-CR. If repeated tPDT was performed more than twice on the same lesion, the lesion L-CR rate was calculated using the evaluable best response in all repeated tPDTs. The L-CR rate by session was calculated as the percentage of sessions that achieved lesion L-CR.

Overall survival (OS) was defined as the time from the date of the first or second tPDT to death. If no events were observed, the interval was censored as the date of the last confirmation of survival. Progression free survival (PFS) was defined as the time from the date of the first or second tPDT to death or recurrent. If no events were observed, the interval was censored as the date of the last confirmation of no recurrence. Survival curves were estimated using the Kaplan–Meier method.

Statistical analyses were performed using JMP® 15 (SAS Institute Inc., Cary, NC, USA).

### Assessment of adverse events

We examined all sessions of repeated tPDT according to local adverse events related to repeated tPDT. According to systemic adverse events related to repeated tPDT, if multiple sessions were performed in a patient at the same time, we examined the multiple sessions as one. Adverse events related to repeated tPDT were assessed according to the Common Terminology Criteria for Adverse Events version 5.0.

## Results

### Patient’s characteristics

**Fig. 1:**
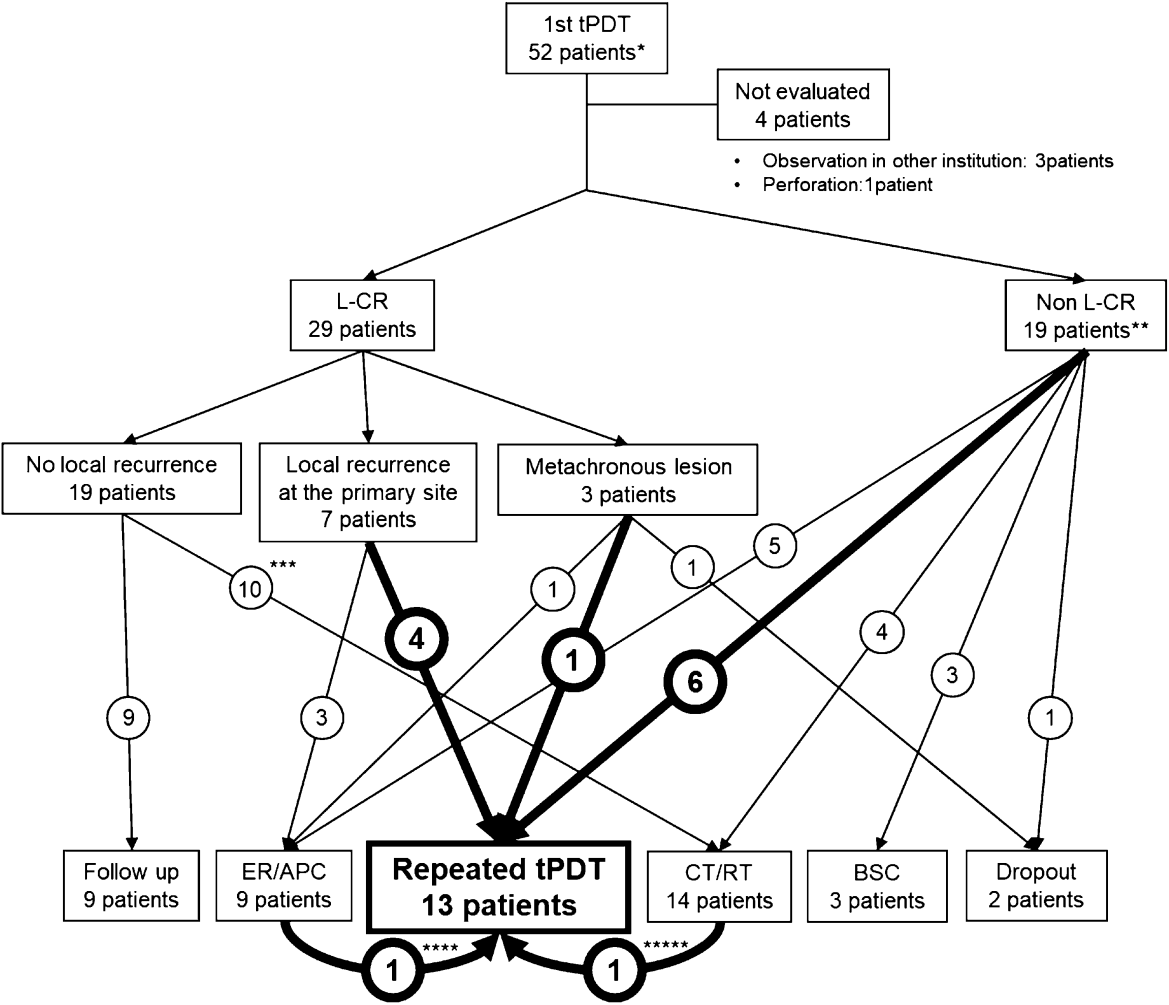
Flow diagram of patients. ER, endoscopic resection; APC, argon plasma coagulation; CT, chemotherapy; RT, radiotherapy; BSC, best supportive care. Primary site means the lesion treated with the first tPDT. * Fifty-one patients treated with the first tPDT involved two patients who had been treated with porfimer sodium photodynamic therapy. **One patient was treated two times with planned tPDT owing to five lesions. *** Four patients were treated with chemotherapy/chemoradiotherapy for lymph node metastasis. Two patients were treated with radiotherapy for brain metastasis. One patient was treated with chemotherapy for intramural metastasis. Three patients were treated with chemotherapy for other cancers. ****One patient who had a residual lesion after the first tPDT was treated with APC followed by repeated tPDT. ***** One patient, who achieved L-CR after the first tPDT, had intramural metastasis, and was then treated with chemotherapy followed by repeated tPDT

Among 52 patients treated with the first tPDT, 29 achieved L-CR, 19 did not achieve L-CR, and four patients were not evaluated (NE) (Fig. 1). Fifty-eight lesions were treated with the first tPDT, of which 35 lesions achieved lesion L-CR, 18 lesions did not achieve L-CR, and five lesions were NE.

**Fig. 2:**
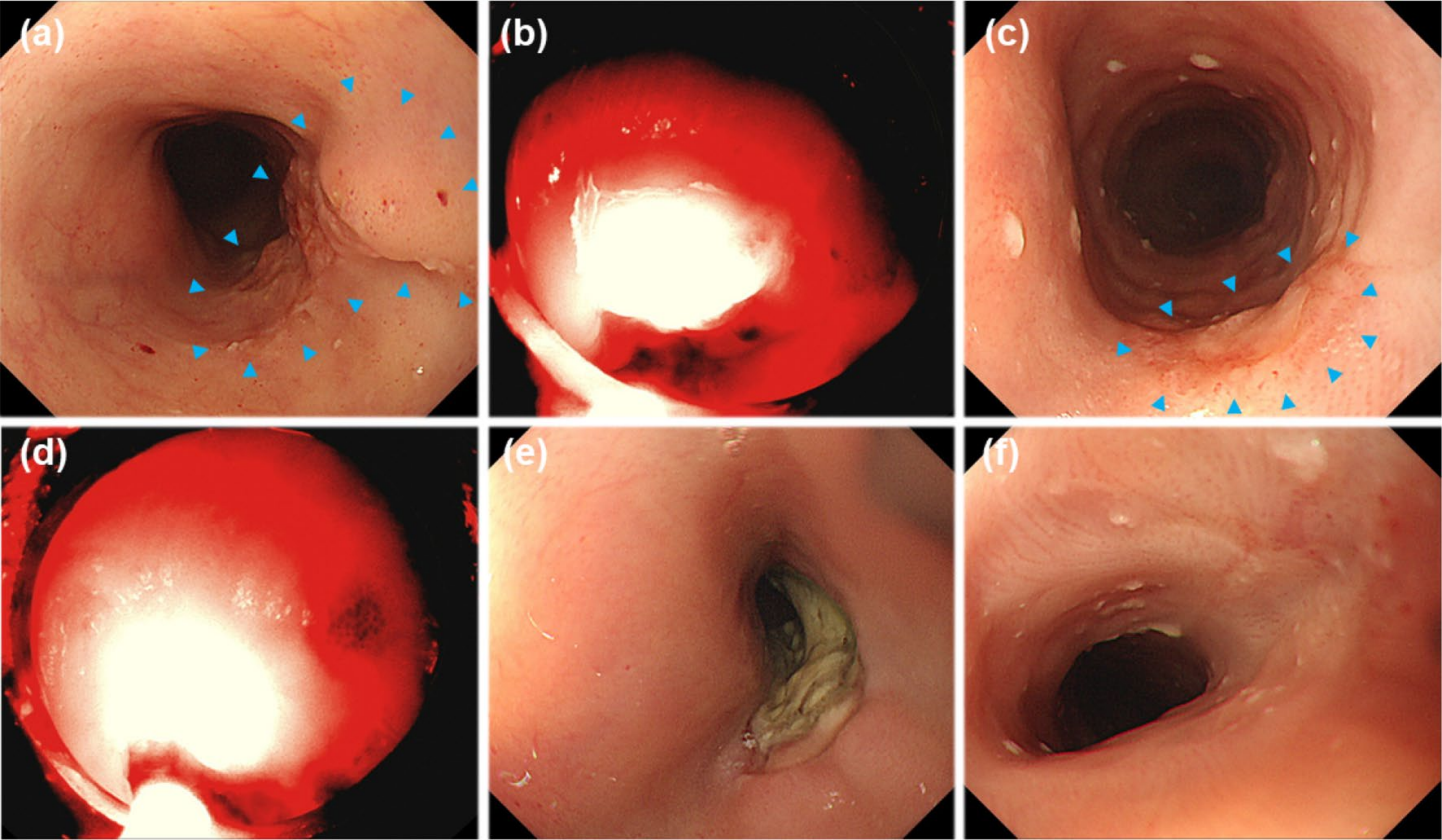
Representative case of local recurrent after the first taraporfin PDT (tPDT). **a** Esophageal cancer was observed in the irradiation field (arrow heads). **b** First tPDT was performed. **c** Recurrent lesion was observed near the scar after achievement of L-CR by the first tPDT. Blue arrowhead means recurrent lesion after the first tPDT. **d** Repeated tPDT was indicated for the recurrent lesion after initial PDT. **e** Ulcer was formed after repeated tPDT. **f** Lesion L-CR was achieved

Among the 52 patients, repeated tPDT after the first tPDT was indicated for 13 patients (25%), of which six had residual tumor, four had local recurrence at the primary site, and six had metachronous lesion (Supplemental Table 1). Figure 2 shows a demonstrable case treated with repeated tPDT owing to recurrence after the first tPDT.

**Fig. 3:**
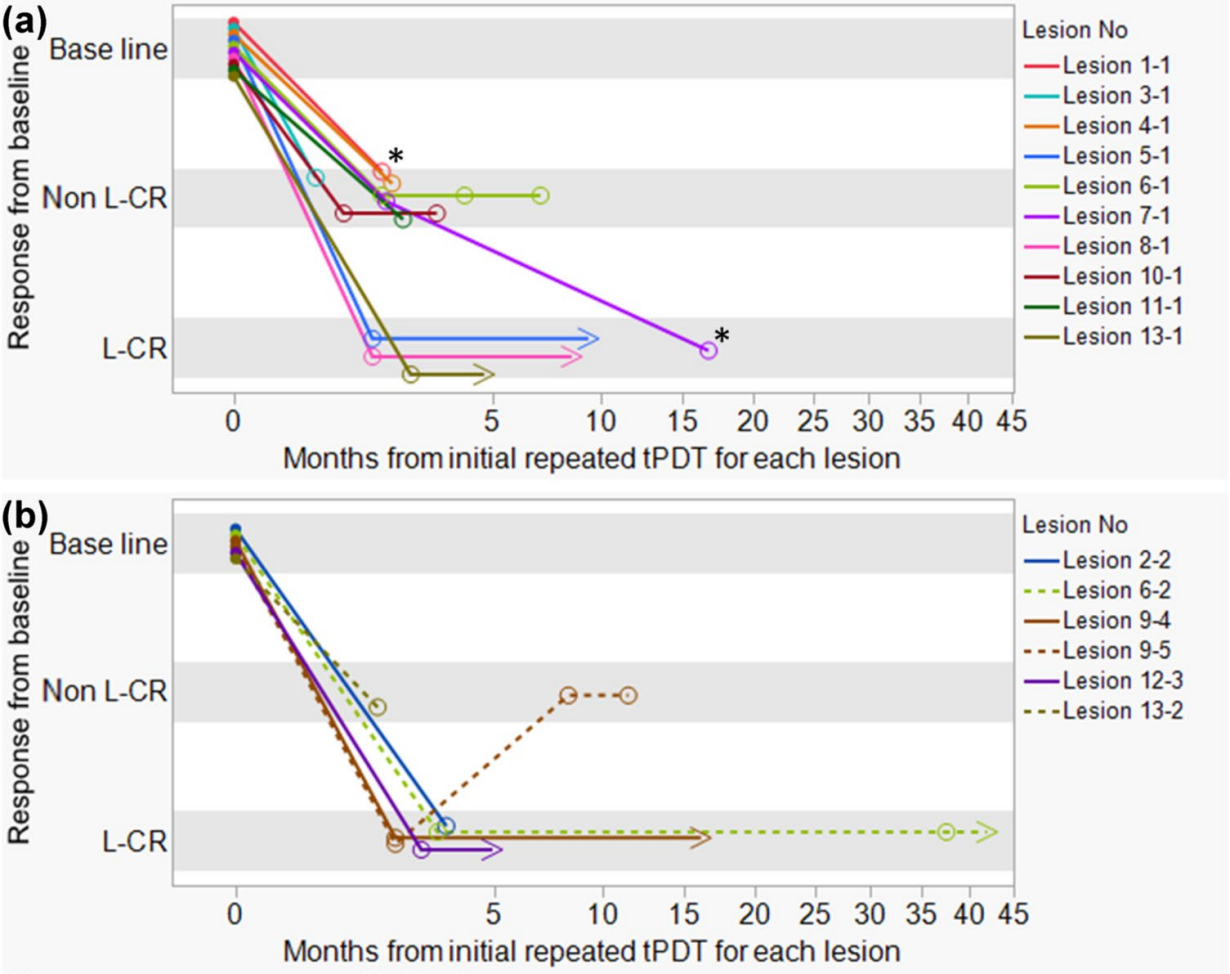
Time course of the response of repeated taraporfin PDT (tPDT). Horizontal axis indicates the month after initial repeated tPDT for each lesion. Zero month means the date of initial repeated tPDT for each lesion. Vertical axis indicates the response to each repeated tPDT. Baseline means the target lesions’ status before repeated tPDT. **a** Time course of the response to repeated tPDT for the primary site. Of the 10 lesions, three lesions achieved L-CR by the second tPDT (Lesion 5–1, 8–1, 13–1). While seven lesions were non L-CR by the second tPDT, one lesion (Lesion 7–1) finally achieved L-CR by the third tPDT. **b** Time course of the response to repeated tPDT for the metachronous site. Of the six lesions, five lesions (Lesion 2–2, 6–2, 9–4, 9–5, 12–3) achieved L-CR by the second or third tPDT (Supplemental Table 1). While two lesions (Lesion 6–2, Lesion 9–5) showed progression after L-CR by the second or third tPDT, one (Lesion 6–2) achieved L-CR again by the repeated tPDT. Primary site means the lesion treated with the first tPDT. Metachronous site means any lesion other than the lesion treated with the first tPDT. Baseline: target lesion status before the initial repeated tPDT for each lesion. Non L-CR: local non complete response, L-CR: local complete response. 〇: best response to repeated tPDT. > : last date of L-CR confirmation. *: Lesion 1–1 and Lesion 7–1 were treated again by repeated tPDT; however, these lesions were not evaluated at timing of this analysis

Table 1 shows the characteristics of 13 patients who were treated with repeated tPDT. In these 13 patients, 16 lesions were indicated for repeated tPDT, 10 lesions were at the primary site treated with the first tPDT, and six lesions were at the metachronous site (Fig. 3). A total of 25 tPDT sessions were performed; 16 sessions were performed at the primary site, and nine sessions were performed at the metachronous site (Supplemental Table 1). Multiple sessions of tPDT on the same lesion were performed in 12 lesions; 10 lesions were at the primary site and two were at the metachronous site.

**Table 1:** Characteristics of the 13 patients

Characteristics	Number of patients
Gender	
Male	10
Female	3
Median age (range)	77 (57–89)
PS	
0	13
T stage before chemoradiotherapy (UICC 8th)	
T1	4
T2	2^a^
T3	4
T4	2
Others	1^b^
Regimen of chemoradiotherapy	
Cisplatin and 5FU and radiotherapy	7
Nedaplatin and 5FU and radiotherapy	1
5FU and radiotherapy	1
Tegafur/Gimeracil/Oteracil and radiotherapy	1
Radiotherapy alone	3^b^
Past porfimer sodium photodynamic therapy	2
Pathology	
Squamous cell carcinoma	12
Adenocarcinoma	1

^a^One patient was treated with the first tPDT for intramural metastasis

^b^One patient was treated with RT for lung cancer, and the first tPDT was performed for esophageal cancer in the irradiation field

### Response rate

Of the 13 patients who were treated with the second tPDT, six (46.2%) achieved L-CR, whereas seven did not (Supplemental Table 1). Of the 16 lesions, nine achieved L-CR (56.3%) by repeated tPDT, whereas seven did not (Fig. 3 and Supplemental Table 1). Figure 3 shows the time course of the response of repeated tPDT. Of the 10 residual/recurrent lesions at the primary site, three lesions achieved L-CR by the second tPDT. While seven lesions were non L-CR by the second tPDT, one lesion could achieve L-CR by the third tPDT. Finally, four achieved L-CR by repeated tPDT. Of the 6 lesions at the metachronous site, five achieved L-CR by repeated tPDT. While two lesions showed progression after L-CR by initial repeated tPDT for each lesion, one achieved L-CR again by the repeated tPDT. Among the 16 lesions, 12 were treated with multiple sessions of tPDT on the same lesion of which five (41.7%) achieved L-CR (Table 2). Four lesions (33.3%) achieved L-CR by the second tPDT for each lesion, one lesion finally achieved L-CR by the third tPDT for each lesion. The total session of repeated tPDT was 25: 10 sessions achieved L-CR (40%), 13 sessions did not achieve L-CR, and two sessions were NE (Supplemental Table 2).

**Table 2:** Lesion status and the best response to repeated tPDT for the same lesion

Lesion no (*n* = 12)	Lesion status before 2nd tPDT for each lesion							
	Location	T stage	Length (mm)	Circumference	Size compared with the lesion size before the initial tPDT for each lesion	Response of 2nd tPDT for each lesion	Total number of tPDT	Best response of repeated tPDT for each lesion
Lesion 1–1	Primary site	TX	10	≤ 1/4	No change or increased	Non L-CR	3	Non L-CR
Lesion 3–1	Primary site	T2	20	> 1/4, ≤ 1/2	No change or increased	Non L-CR	2	Non L-CR
Lesion 4–1	Primary site	T1	12	> 1/4, ≤ 1/2	No change or increased	Non L-CR	2	Non L-CR
Lesion 5–1	Primary site	T1	4	≤ 1/4	Decreased	L-CR	2	L-CR
Lesion 6–1	Primary site	T1	5	≤ 1/4	Decreased	Non L-CR	4	Non L-CR
Lesion 6–2	Metachronous site	T2	30	> 1/4, ≤ 1/2	No change or increased	L-CR	2	L-CR
Lesion 7–1	Primary site	T2	30	> 1/4, ≤ 1/2	Decreased	Non L-CR	4	L-CR
Lesion 8–1	Primary site	T1	10	≤ 1/4	Decreased	L-CR	2	L-CR
Lesion 9–5	Metachronous site	T1	10	≤ 1/4	No change or increased	Non L-CR	3	Non L-CR
Lesion 10–1	Primary site	T1	15	≤ 1/4	No change or increased	Non L-CR	3	Non L-CR
Lesion 11–1	Primary site	T1	10	> 1/4, ≤ 1/2	No change or increased	Non L-CR	2	Non L-CR
Lesion 13–1	Primary site	T2	20	≤ 1/4	Decreased	L-CR	2	L-CR
Lesion L-CR rate						33.3% (4/12)		41.7% (5/12)

### Overall survival and progression free survival

**Fig. 4:**
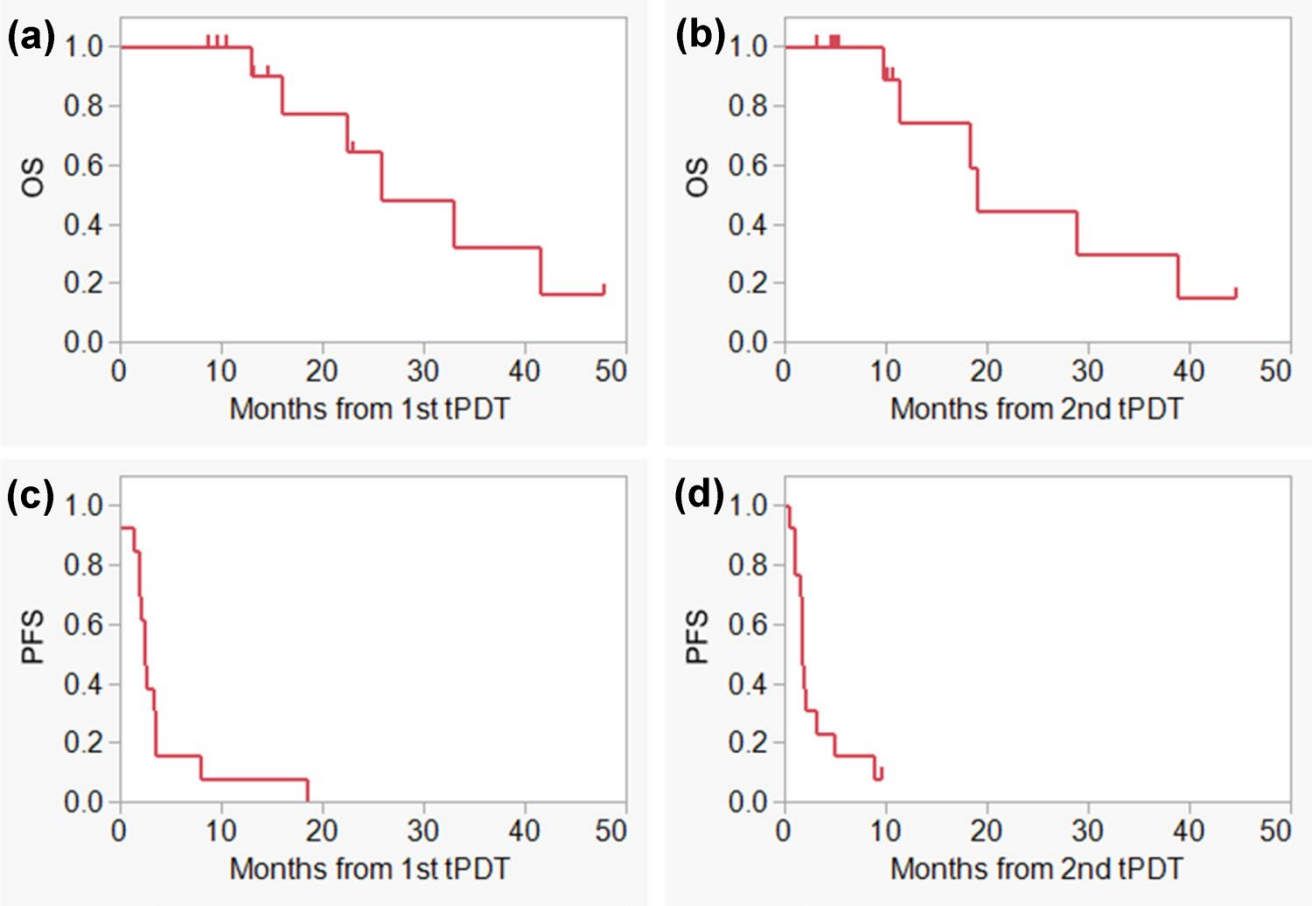
Overall survival and progression free survival after tPDT. **a** OS from the date of the first tPDT, **b** OS from the date of the second tPDT, **c** PFS from the date of the first tPDT, **d** PFS from the date of the second tPDT

During the median follow-up period of 15.9 months (range 8.7–47.8), six patients died. The median OS from the first and second tPDT were 25.8 and 19.1 months, respectively, and the median PFS from the first and second tPDT were 2.4 and 1.7 months, respectively (Fig. 4).

### Adverse events

We examined 25 sessions of repeated tPDT in 13 patients according to local adverse events related to tPDT. In two patients, two sessions were performed in each patient at the same time. Therefore, we examined 23 sessions based on systemic adverse events. Five sessions (24%) developed esophageal stenosis (Table 3). There were no severe local adverse events except in one patient. This patient (4%) developed grade 3 esophageal stenosis and grade 3 esophageal perforation. Ten sessions (43.5%) developed esophageal pain, nine sessions (39.1%) developed nausea, and five sessions (21.7%) developed fever. None of the patients developed grade 3 or higher systemic adverse events involving photosensitivity. No treatment-related deaths occurred.

**Table 3:** Adverse events related to repeated tPDT

Local adverse events (25 sessions)	CTCAE_v5.0 grade				Total (%)	Rate of grade 3–4 (%)
	1	2	3	4		
Esophageal hemorrhage	0	0	0	0	0	0
Esophageal stenosis	2 (8.0%)	3 (12.0%)	1 (4.0%)	0	6 (24.0%)	1 (4.0%)
Esophageal perforation	0	0	1 (4.0%)	0	1 (4.0%)	1 (4.0%)

Systemic adverse events (23 sessions)	CTCAE_v5.0 grade				Total (%)	Rate of grade 3–4 (%)
	1	2	3	4		
Esophageal pain	10 (43.5%)	0	0	0	10 (43.5%)	0
Nausea	9 (39.1%)	0	0	0	9 (39.1%)	0
Fever	5 (21.7%)	0	0	0	5 (21.7%)	0
Skin photosensitivity	0	0	0	0	0	0
AST increased	3 (13.0%)	0	0	0	3 (13.0%)	0
ALT increased	1 (4.3%)	0	0	0	1 (4.3%)	0
Blood bilirubin increased	0	0	0	0	0	0

## Discussion

Salvage tPDT is an indispensable treatment for patients who have local failure after CRT for esophageal cancer. However, an effective treatment for local failure after the first tPDT has not been established. Previously, salvage surgery, ER, and chemotherapy have indicated local failure after porfimer sodium photodynamic therapy [16]. However, safety and efficacy of repeated porfimer sodium photodynamic therapy has not been elucidated. In this study, we analyzed the 13 patients with local failure after the first tPDT who underwent repeated tPDT, and we showed that repeated tPDT was safe and effective.

In our prospective study, the L-CR and lesion L-CR rates of the first tPDT for local failure of esophageal cancer treated with CRT were 88.5% and 89.3%, respectively [14]. In practice, a recent retrospective study by a single institute reported that the L-CR rate of first tPDT was 69% [15]. In this study, the L-CR rate of repeated tPDT was 46.2%, and the lesion L-CR rate was 56.3%. In particular, the lesion L-CR rate of repeated tPDT on the same lesion was 41.7%. Good control of local lesions by repeated tPDT might contribute to improving the prognosis of patients who experienced local failure after the first tPDT. In fact, the median OS from second tPDT was 19.1 months. These results indicated that repeated tPDT was an effective treatment option with the possibility of treating patients who have no surgical and ER indications.

As for the efficacy of repeated tPDT on the same lesion, out of the five lesions whose size before the second tPDT for each lesion was decreased compared with the lesion size before the initial tPDT for each lesion, four lesions (80%) achieved L-CR by repeated tPDT. Out of the seven lesions whose size was unchanged or increased compared with the lesion size before the initial tPDT for each lesion, only one lesion (14.3%) achieved L-CR by repeated tPDT. These results indicated that it is difficult to expect a sufficient effect of repeated tPDT in lesions that did not respond or quickly re-increased after the initial tPDT. Therefore, to achieve L-CR by repeated tPDT, it is important to find residual or recurrent lesions, while they are still small, and repeated tPDT should be administered as soon as possible. In our institution, endoscopic surveillance is performed every 2–3 weeks after tPDT until healing is confirmed, and every month from healing to 6 months after PDT. Frequent endoscopic surveillance lead to early detection of residual tumor or local recurrence and provide opportunities to achieve L-CR by repeated tPDT.

To evaluate the safety of repeated tPDT, we assessed major adverse events, such as esophageal pain, fever, esophageal stenosis, photosensitivity, and liver dysfunction. In the first tPDT for esophageal cancer, esophageal pain, fever, esophageal stenosis, skin photosensitivity, increased AST, increased ALT, and increased blood bilirubin levels were reported in 53.8%, 30.8%, 7.7%, 0%, 19.2%, 19.2%, and 7.7% of patients, respectively [14]. Other studies reported that the frequencies of esophageal stenosis, esophageal perforation, and skin photosensitivity were 4.5–6.3%, 0%, and 0–4.5%, respectively [15, 17]. In this study, grade 4 or higher adverse events related to repeated tPDT were not observed in any patient, and there were no treatment-related deaths. Repeated administration of talaporfin sodium at adequate intervals did not worsen the risk of skin photosensitivity and serious liver damage. Grade 3 esophageal stenosis and grade 3 esophageal perforation were observed in a patient who had previously undergone four sessions of porfimer sodium photodynamic therapy and was treated with repeated tPDT three times on the same lesion. In other patients who had undergone repeated tPDT more than three times on the same lesion, severe esophageal stenosis and perforation were not observed. Repeated treatment with porfimer sodium photodynamic therapy in the past may have increased the risk of esophageal stenosis and perforation. Nevertheless, repeated tPDT for the same lesion was thought to cause esophageal stenosis and esophageal perforation. To reduce the risk of severe stenosis or perforation, we use an endoscopic hood tailored for PDT to prevent scattered laser light from hitting areas outside the target area, and avoid circumferential laser irradiation. In addition, the total energy of laser irradiation is basically not to exceed 700 J. In patient with severe stenosis, we try to improve the stenosis by careful endoscopic dilatation as needed. Patient with perforation is basically managed with central venous nutrition and wait for the perforation to close.

This study had some limitations. First, the sample size was small. Second, the confirmation of histological L-CR was unknown in some lesions owing to observation in other hospitals or other reasons. Third, in this study, the criteria of L-CR were defined as the disappearance of the lesion at least once for some reasons; therefore, a few lesions recurred immediately after achievement of L-CR.

In conclusion, repeated tPDT is an effective and safe treatment option for local failure after salvage tPDT for esophageal cancer.

## Supplementary Information

Below is the link to the electronic supplementary material.

Supplementary file1 (DOCX 87 KB)
