# Supplementary material for: Repeated talaporfin sodium photodynamic therapy for esophageal cancer: safety and efficacy
Source: Esophagus. 2021 Jun 9;18(4):817–24. doi: 10.1007/s10388-021-00853-x (PMC8387249; doi:10.1007/s10388-021-00853-x)

Supplemental Table 1 Target for the repeated tPDT and response for tPDT of each lesion


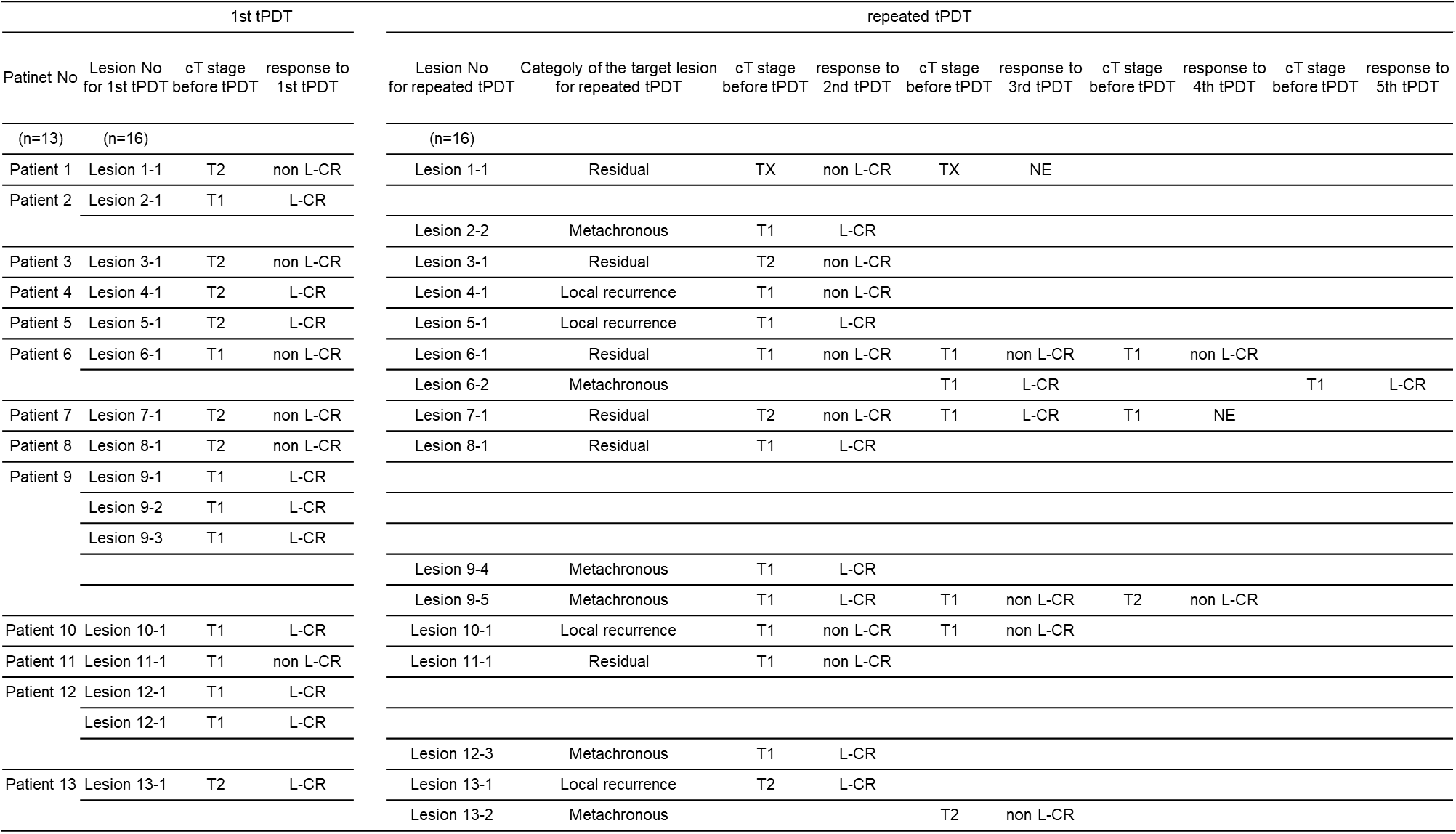


Supplemental table 2: L-CR rate of repeated tPDT by session


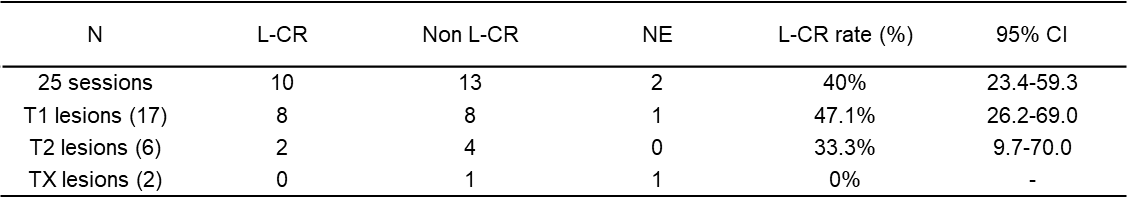

Supplement: Supplementary file 1 — Supplementary file1 (DOCX 87 KB) [file 10388_2021_853_MOESM1_ESM.docx]
